# Record high *T*_c_ element superconductivity achieved in titanium

**DOI:** 10.1038/s41467-022-33077-3

**Published:** 2022-09-15

**Authors:** Changling Zhang, Xin He, Chang Liu, Zhiwen Li, Ke Lu, Sijia Zhang, Shaomin Feng, Xiancheng Wang, Yi Peng, Youwen Long, Richeng Yu, Luhong Wang, Vitali Prakapenka, Stella Chariton, Quan Li, Haozhe Liu, Changfeng Chen, Changqing Jin

**Affiliations:** 1 Beijing National Laboratory for Condensed Matter Physics, Institute of Physics, Chinese Academy of Sciences, 100190 Beijing, China; 2 School of Physical Sciences, University of Chinese Academy of Sciences, 100190 Beijing, China; 3 Songshan Lake Materials Laboratory, 523808 Dongguan, China; 4 International Center for Computational Method and Software, College of Physics, Jilin University, 130012 Changchun, China; 5 Shanghai Advanced Research in Physical Sciences, 201203 Shanghai, China; 6 Center for Advanced Radiations Sources, University of Chicago, Chicago, IL 60637 USA; 7 Center for High Pressure Science & Technology Advanced Research, 100094 Beijing, China; 8 Department of Physics and Astronomy, University of Nevada, Las Vegas, NV 89154 USA

**Keywords:** Superconducting properties and materials, Electronic properties and materials

## Abstract

It is challenging to search for high *T*_c_ superconductivity (SC) in transition metal elements wherein *d* electrons are usually not favored by conventional BCS theory. Here we report experimental discovery of surprising SC up to 310 GPa with *T*_c_ above 20 K in wide pressure range from 108 GPa to 240 GPa in titanium. The maximum *T*_c_^onset^ above 26.2 K and zero resistance *T*_c_^zero^ of 21 K are record high values hitherto achieved among element superconductors. The *H*_c2_(0) is estimated to be ∼32 Tesla with coherence length 32 Å. The results show strong *s*-*d* transfer and *d* band dominance, indicating correlation driven contributions to high *T*_c_ SC in dense titanium. This finding is in sharp contrast to the theoretical predications based on pristine electron-phonon coupling scenario. The study opens a fresh promising avenue for rational design and discovery of high *T*_c_ superconductors among simple materials via pressure tuned unconventional mechanism.

## Abstract (web summary)

Superconductivity at megabar pressures has recently attracted interest in the context of hydrides. Here, the authors demonstrate superconductivity up to 26 K at high pressure in elemental titanium, and further suggest that electron correlations contribute to the high *T*_c_.

## Introduction

Titanium (Ti) metal has long attracted tremendous scientific interests because of its combined properties of light weight, high strength and corrosion resistance. As an advanced metallic structural material, Ti and its alloys find wide use in the fields of aerospace, biomedicine and at extreme conditions^1–3^. High pressure can modify crystal structures which, in turn, may lead to new functionalities. At ambient pressure and room temperature, Ti crystalizes in a hexagonal close-packed (hcp) structure (Ti-α phase)^4^. Under applied pressure, Ti undergoes structural transitions in the sequence of Ti-α, Ti-ω, Ti-γ, Ti-δ, and Ti-β phases, where Ti-ω phase is a hexagonal structure, Ti-γ and Ti-δ phases are orthorhombic and Ti-β phase is body-centered cubic^5–9^. The α-to-ω transition occurs around 8 GPa^5,6^, and the Ti-ω phase is stable up to about 100 GPa, then transforms into Ti-γ phase^6,10^, which further transforms into Ti-δ phase at ~140 GPa^6^, before cubic Ti-β phase stabilizes at 243 GPa^9^.

Superconductivity (SC) in high-pressure phases of Ti metal was previously reported to have a measured maximal critical temperature (*T*_c_) of 3.5 K at 56 GPa^11^; early theoretical calculations based on the electron-phonon coupling mechanism predicate that the *T*_c_ for Ti metal is capped at about 5 K for all the known high-pressure phases^12^. Generally, compression of crystal lattice has markedly different effects on the 4*s* and 3*d* bands, prompting notable *s*-*d* electron transfer. The narrower *d* bands possess stronger correlation characters, while the *s-d* transfer tends to enhance electronic density of state (DOS) near the Fermi level in favor of SC^13–17^. Here, we report a surprising experimental observation of dramatic pressure enhanced SC in Ti over a wide pressure range with the maximal *T*_c_ above 26 K, setting a new record among element superconductors. Measured normal state conductivity results and analysis of electronic and superconducting properties indicate prominent influence of the *s-d* transfer and *d*-band driven correlation effects on the significantly enhanced and unusually high *T*_c_ SC in highly compressed Ti metal.

## Results

### High-pressure superconductivity at high pressure

**Fig. 1:**
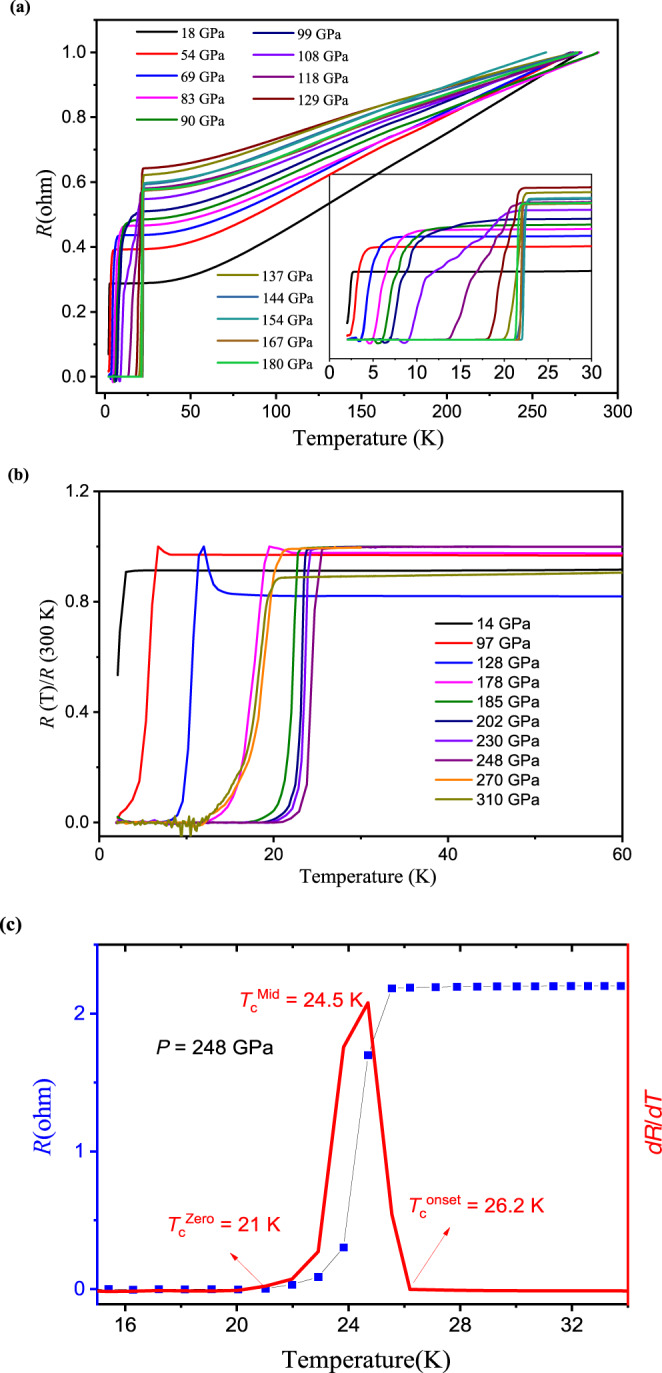
The superconductivity measurements. **a** Temperature dependence of the electrical resistance of elemental metallic Ti (Sample 1) measured at high pressures. The inset is an enlarged view of the resistance curve, showing the superconducting transition in detail. **b** The resistance curves for Ti Sample 2. **c** The resistance curve measured at 248 GPa, where the derivative of the resistance with respect to temperature *dR*/*dT* is plotted to clearly show the onset *T*_c_.

The high-pressure resistance experiments have been carried out using diamond anvil cell apparatus. The sample assembly and the electrode configuration for four probe Van der Pauw method are shown in Supplementary Fig. 1a, b. The pressure is calibrated by the shift of the first-order Raman edge frequency from the diamond cutlet as shown in Supplementary Fig. 1c. Figure 1a shows the experimentally measured temperature dependence of electrical resistance at high pressure up to 180 GPa for Ti metal in Sample 1. The results show that Ti metal becomes superconductive with *T*_c_ above 2 K at 18 GPa and rises slightly to ~3.5 K at 54 GPa, which agrees with previously reported results^11^. The value of *T*_c_ increases at an enhanced pace from 54 to 99 GPa, then undergoes a steep rise from 10.2 K at 99 GPa to 20.3 K at 108 GPa, and *T*_c_ is further enhanced to 22 K at 180 GPa. For Sample 2, the pressure range was increased, with the highest pressure reaching 310 GPa that was calibrated by using the method described in ref. 18. The resistance curves at pressure are shown in Fig. 1b. With further increasing pressure the *T*_c_ reaches a maximum of 26 K at 248 GPa, as shown in Fig. 1c. The onset critical temperature (*T*_c_^onset^), the midpoint critical temperature (*T*_c_^mid^) and the zero resistance critical temperature (*T*_c_^zero^) of superconducting transition are determined by the derivative of resistance with respect to temperature d*R*/d*T* as shown in Fig. 1c. It is noted that *T*_c_ stays almost constant over a wide pressure range up to at least 240 GPa, and such robust superconducting behaviors are consistently seen in different specimens (Supplementary Fig. 2). At 108 GPa where *T*_c_ jumps up, the resistance exhibits a two step superconducting transition behavior with the lower *T*_c_ of ~11 K that is comparable to the *T*_c_ value at 99 GPa, indicating a phase transition near 108 GPa, which is close to the pressure for the reported ω-γ phase transition^6,10^. In the Ti-ω phase, *T*_c_ rises smoothly with pressure below 56 GPa with a slope of 0.07 K/GPa; assuming this slope keeps unchanged, *T*_c_ should reach 8.7 K before the ω-γ phase transition (at 128 GPa)^11^. However, our results show that the slope d*T*_c_/d*P* increases significantly between 54 and 99 GPa, which leads to the much higher measured *T*_c_ at 99 GPa.

**Fig. 2:**
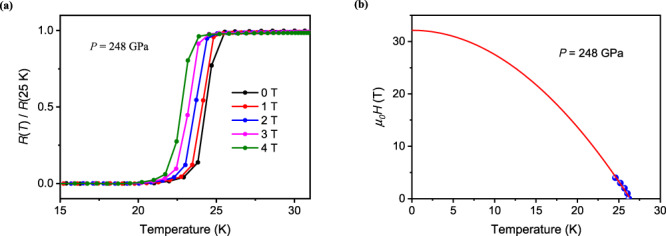
The superconductivity at magnetic field. **a** Temperature dependence of the electrical resistance of Ti metal measured at different magnetic fields at the fixed pressure of 248 GPa. **b** Upper critical field versus superconducting transition temperature of *T*_c_^zero^. The line is a fit obtained using the Ginzburg–Landau function.

To further probe the pressure driven SC phase of Ti metal, we have examined the effect of magnetic field on the SC transition behavior. Figure 2a presents the electrical resistance measured at 248 GPa and at different magnetic fields. It is seen that the transition is gradually suppressed by the magnetic field. We plotted onset *T*_c_ versus magnetic field in Fig. 2b, from which the upper critical field at zero temperature *μ*_0_*H*_c2_(0) can be estimated. The *μ*_0_*H*_c2_(*T*) date were fitted to the Ginzburg Landau (GL) function,

1 $${{\mu }_{0}H}_{{{{{{\rm{c}}}}}}2}\left(T\right)={{\mu }_{0}H}_{{{{{{\rm{c}}}}}}2}\left(0\right)(1-{(T/{T}_{{{{{{\rm{c}}}}}}})}^{2})$$

which gives a value of *μ*_0_*H*_c2_(0) = 32 T. At other pressures where *T*_c_ is above 20 K, the estimated upper critical field has been obtained to be near 30 T (Supplementary Fig. 3a, b), which is larger than that of the most commonly used low-temperature NbTi superconductor (*μ*_0_*H*_c2_(*T*) = 15 T). Using the *μ*_0_*H*_c2_(*T*) value of 32 T, the GL coherence length was calculated to be ξ = 32 Å via *μ*_0_*H*_*c*2_(0)= Φ_0_/2πξ^2^, where Φ_0_ = 2.067 × 10^−15^ Web is the magnetic flux quantum.

### Superconducting phase diagram

**Fig. 3:**
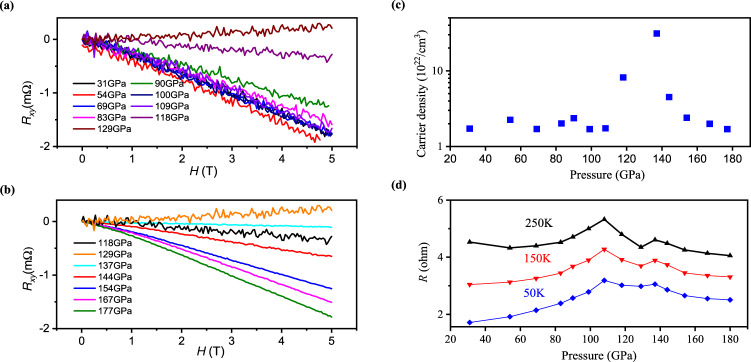
The Hall measurements. **a**, **b** Hall resistance as a function of magnetic field measured at different pressure. **c**, **d** Carrier density and resistance at fixed temperature versus pressure, respectively.

Figure 3a, b present the measured Hall resistance at room temperature and different pressures. The Hall resistance is negative and decreases linearly with magnetic field, indicating that the major carriers are electrons. The carrier density *n* as a function pressure estimated from the Hall resistance is presented in Fig. 3c, with a value of about 2.5 × 10^22^/cm^3^ that is little changed in the low-pressure range. Above 108 GPa, *n* increases dramatically and is enhanced by more than one order of magnitude to 3.1 × 10^23^/cm^3^ at 137 GPa. Further increasing pressure leads to reduced carrier density of 4.5 × 10^22^/cm^3^ at 144 GPa. The changes of the carrier density indicate phase transitions at pressures of about 108 and 144 GPa, respectively. To see more clearly these phase transitions, the pressure dependence of resistance *R*(*P*) at fixed temperature is plotted in Fig. 3d. The *R*(*P*) curve shows two peaks near the critical pressures, which corresponds to the ω–γ phase and γ–δ phase transitions reported by the authors in refs. 6, 10, respectively.

**Fig. 4:**
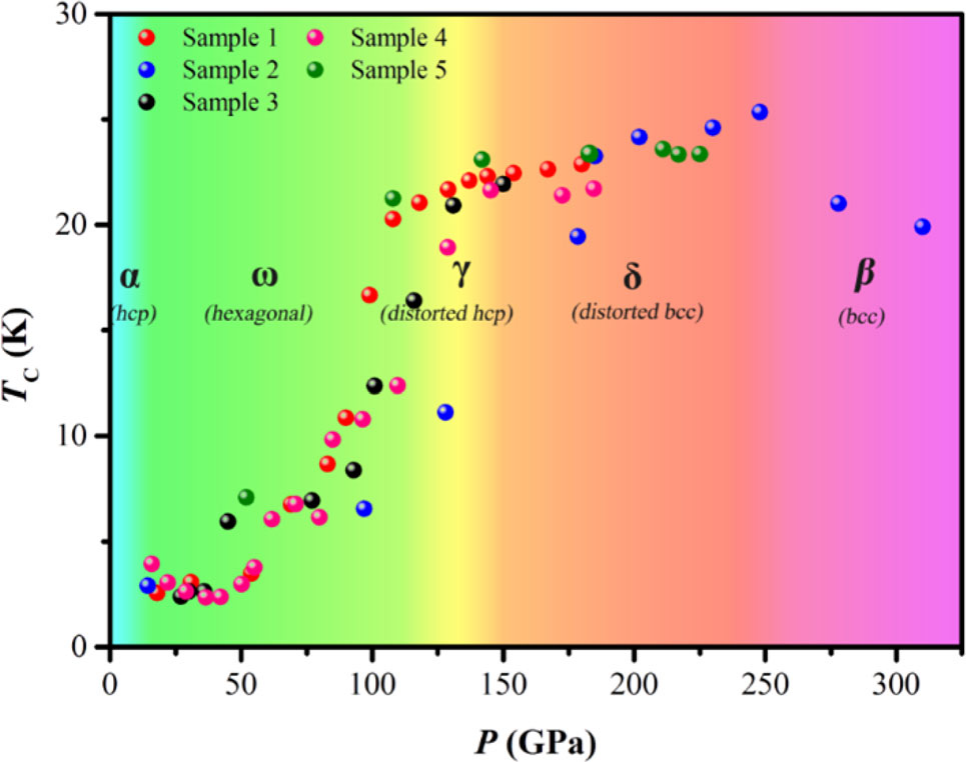
Superconducting phase diagram. The superconducting critical transition temperature (*T*_c_) of Ti metal at compression up to 320 GPa versus high-pressure phases.

We have carried out the high-pressure X ray diffraction experiments, and the results are shown in Supplementary Fig. 4a–d. Combining the phase transition reported by previous works and our transport experiments, we plot the superconducting versus structural phase diagram in Fig. 4. Up to 310 GPa, five different crystal structures, in the sequence of Ti-α, Ti-ω, Ti-γ, Ti-δ, and Ti-β are identified. The Ti-α phase (*P* = 0–9 GPa) hosts SC with *T*_c_ below 2 K; the Ti-ω phase (*P* = 9–108 GPa) sees a monotonously increasing *T*_c_ with pressure to 12 K at ~108 GPa; while in the high-pressure phases of Ti-γ (*P* = 108–144 GPa) and Ti-δ (*P* = 144–240 GPa), *T*_c_ stays above 20 K with the maximum *T*_c_ = 26 K occurring at the boundary of Ti-δ and Ti-β phases. It is remarkable that the *T*_c_ of compressed Ti metal stays robust above 20 K in a very wide range of pressures. This behavior is at odds with the expectation of conventional phonon-mediated superconducting theory, which predicts sensitive pressure dependence and descending *T*_c_ at very high pressures due to phonon stiffening. Interestingly, it is noted that the phenomenon of robust *T*_c_ values extended over a wide pressure range has been found in high-*T*_c_ cuprates wherein SC is driven by correlation effects^19^. This analogy indicates that mechanisms of novel many-body origin may be driving the unexpectedly SC in dense Ti metal at ultrahigh pressures.

## Discussion

Among all elements, only a few have been reported to exhibit SC with *T*_c_ near 20 K at high pressures, including alkali metal Li^20,21^, alkali earth metal Ca^15^ and rare earth metals of Y^16^. The high *T*_c_ values of Li, which is the lightest element metal containing only simple 2*s* electrons, is attributed to the enhancement of the electron-phonon coupling due to the phonon modes softening under high pressure^22^; while the *T*_c_ enhancements in Ca and Y are mainly ascribed to pressure induced electron transfer from the *s* to *d* shell. The superconducting Ti at high pressure has the highest *T*_c_ among the elements in the period table.

The resistance of Ti as a function of temperature measured at 18 GPa (Supplementary Fig. 5) exhibits interesting scaling behavior. Upon fitting with the formula *R* = *R*_0_ + A*T^*n*^, where *R*_0_ is the residual resistance, *A* is the coefficient of the power law, and *n* is the exponent, the resulting scaling exponent *n* = 3.1 is different from the value expected for systems dominated by either electron-phonon scattering (*n* = 5) or pure electron-electron scattering (*n* = 2). In fact, the *n* value near 3 implies that the *s*–*d* interband scattering dominates the electron transport with contributions from electron correlation effects, as observed in 1T-TiSe_2_^23^ or Ta_4_Pd_3_Te_16_^24^. It reveals that at 18 GPa the energies between 4*s* and 3*d* shells are very near to each other and their electron configurations are mixed. Upon further applying pressure, the *s* band will move to higher energy as shown in the following calculations, promoting the *s*-*d* electron transfer, increasing the number of *d* electrons and inducing sequence of structural phase transitions. As a result, the *T*_c_ is greatly enhanced and a record *T*_c_ among the elements has been achieved. It seems that the *T*_c_ is closely related with the *s*-*d* electron transfer, which suggests the impact of the *d* electron correlation effects on the formation of Cooper pairs.

We have examined relative energetic stability of the high-pressure phases of Ti metal from first principles calculations, and the results are in generally consistent with the experimentally measured phase stability and transformation sequence. Adopting the determined crystal structures of the Ti metal phases, we have calculated their electronic, phonon and electron-phonon coupling properties, which are used as input to determine the SC critical temperature *T*_c_. The obtained *T*_c_ data for the Ti-ω, Ti-γ, and Ti-δ phases (Supplementary Fig. 6) reveal the following trends: (i) *T*_c_ increases with rising pressure monotonically in the Ti-ω phase over its entire stability range; (ii) *T*_c_ is significantly enhanced upon the phase transition into the Ti-γ phase over the relatively narrow pressure range where this transition occurs; (iii) *T*_c_ undergoes an even larger jump when the structure enters the Ti-δ phase. These findings are in overall agreement with the experimental results up to about 160 GPa, but large discrepancies exist at higher pressures where the record-setting *T*_c_ is observed.

**Fig. 5:**
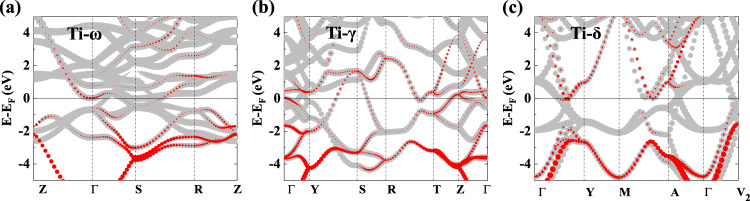
Calculated electronic band structures. **a** Ti-ω at 20 GPa, **b** Ti-γ at 100 GPa, and **c** Ti-δ at 180 GPa. The contributions from the *s* electron and *d* electron states are shown by red and gray circles, respectively, and the circle areas are proportional to the weights of the corresponding band states. Energy is measured relative to the Fermi energy *E*.

The experimentally observed superconducting properties of the densely compressed Ti metal indicate clear inadequacy of the conventional phonon-mediated SC mechanism for describing the unexpectedly high values of *T*_c_ and anomalously robust superconducting state over a very wide pressure range. The electron-electron correlation effects associated with the *d* bands in Ti are expected to have a strong influence on transport and superconducting properties. Calculated electronic band structure of Ti-ω phase at 20 GPa (Fig. 5a) shows significant overlap of the *s* and *d* electron states near the Fermi level, which corroborates our measured normal state resistivity results (Supplementary Fig. 5) indicating strong *s*-*d* scattering contribution to the resistivity^24,25^. At higher pressure of 100 GPa, the band structure of Ti-γ phase (Fig. 5b) shows that the *s* states move up in energy while still have large overlap with the *d* bands, which is conducive to significant *s-d* scattering. At further increased pressure of 180 GPa, the electronic states in Ti-δ phase (Fig. 5c) near the Fermi level are dominated by the *d* electron states. There are flat *d* bands below the Fermi level that can host significant correlation effects. These *d* bands will likely rise much closer to the Fermi level due to the electron-electron interactions when properly treated by many-body theory. Such correlation effects could greatly enhance the *d* band derived electronic DOS at the Fermi level favorable for increasing *T*_c_ and also drive additional non-phonon-mediated mechanisms for further strengthening the superconducting state with higher *T*_c_ and maintaining its robust presence over a wide pressure range. This study raises intriguing possibility of major impact by many-body effects on SC in dense Ti, which needs in-depth study by sophisticated many-body theoretical treatments to explore pertinent novel processes and underlying mechanisms.

The present study unveils unexpectedly high *T*_c_ SC in compressed Ti metal with record-setting among elemental superconductors. The *T*_c_ and *μ*_0_*H*_c2_ values of Ti are notably higher than those of the widely used superconducting NbTi alloy. Our discovery raises the possibility of finding more materials via pressure driven correlation effects stemming from the contributions of *d* electrons, leading to SC with much higher *T*_c_ than previously believed achievable, and such materials may be stabilized at lower pressures via mechanical strain or chemical pressure. This intriguing scenario calls for further research into the impact on SC by the *s*–*d* interaction and *d* electron dominated correlation effects in highly compressed *d* band metals for evaluation of diverse materials, from elemental solids to alloys, in search of hitherto unknown and unexplored superconducting materials that could improve fundamental understanding of broader varieties of superconductors. Equally important, the present findings open new avenues for expanding the scope of superconductors with notably enhanced *T*_c_ and *μ*_0_*H*_c2_ that are more adaptive and suitable for applications in diverse and demanding implementation environments. We became aware during writing the paper (arXiv:2112.12396) that an independent work by Liu et al. was carried out where maximum Tc 23.6K was observed at 145 GPa within experimental pressure up to 183 GPa.^25^.

## Methods

### High-pressure measurements

The electrical resistance and Hall Effect measurements were performed using the four probe Van der Pauw method for tiny specimen as described in Supplementary Methods. The pressures are calibrated via the shift of the first order Raman edge frequency from the diamond cutlet as shown in Supplementary Fig. 1^18^. The applied current is 100 μA. Diamond anvil cells were used to produce high pressures. A variant of anvils with beveled culet size of 20/140/300 μm, 30/140/300 μm or 50/140 300 μm are adopted in the experiments. A plate of T301 stainless steel that is covered with mixture of cBN powder and epoxy as insulate layer was used as the gasket. A hole of approximately 15–30 μm in diameter depending on top culet size was drilled in the center of the gasket to serve as high-pressure chamber. The hBN powder was generally used as pressure transmitting medium that filled in the high-pressure chamber. We used the ATHENA procedure to produce the specimen assembly^26^. Four Pt foils with thickness approximately 0.5 μm as the inner electrode were deposited on the culet surface. Cross shaped Ti specimens with side lengths ~10 μm × 10 μm and thickness of 1 μm were stacked on the electrodes. Pressure was calibrated by the shift of the first order Raman edge frequency from the diamond cutlet^18^. Diamond anvil cells were put into a MagLab system that provides synergetic extreme environments with temperatures from 300 to 1.5 K and magnetic fields up to 9 T for the transport measurements^26–29^.

### High-pressure synchrotron X-ray experiments

In situ high-pressure angle-dispersive X-ray diffraction data were collected at room temperature at GSECARS of Advanced Photon Source at the Argonne National Laboratory. The X-ray with the wavelength *λ* = 0.3344 Å was focused down to a spot of ~3 μm in diameter on the sample. A symmetric diamond anvil cell with beveled anvil (50/300 μm) was used. Rhenium steel gasket was pre-pressed to a thickness of 20 μm, and a hole of diameter of 15 μm was drilled at the center to serve as sample chamber, which was then filled with Ti power mixed with Pt. Pressure was calibrated using the equitation of state of both Re and Pt. The X-ray diffraction images are converted to two dimensional diffraction data with Dioptas^30^.

### First-principle theoretical calculation

To assess structural, electronic, and phonon-mediated superconducting properties of Ti metal under pressure, we employed the latest computational techniques to determine the total energy, lattice dynamics and electron-phonon coupling using the QUANTUM ESPRESSO code^31^, with improved description over previously reported results^12^. Superconducting critical temperature *T*_c_ has been evaluated based on the Eliashberg theory of SC^32,33^, using the following formula that McMillan derived^34^ and later modified by Allen and Dynes^35^,

$${T}_{{{{{{\rm{c}}}}}}}=\frac{{\omega }_{{\log }}}{1.20}\,{\exp }\,\left[-\frac{1.04\,\left(1+\lambda \right)}{\lambda -{\mu }^{*}\left(1+0.62\lambda \right)}\right]$$

where *ω*_log_ is a logarithmically averaged characteristic phonon frequency, and μ^*^ is the Coulomb pseudopotential, which describes the effective electron–electron repulsion^36^. This equation is generally accurate for materials with EPC parameter *λ* at 1.5 or less ^37,38^, which is satisfied in the present study. The Coulomb pseudopotential μ^*^ is often treated as an adjustable parameter with values within a narrow range around 0.1 for most materials, making this formulism highly robust^36–39^, and compares well with the latest ab initio Eliashberg theory. In this work, the commonly used value of μ^*^ = 0.13 is adopted for all the reported calculations. Such pseudopotential calculations have been employed to study structural stability and transformation of Ti compressed up to at least 200 GPa, and the results are in good agreement with those from full potential calculations and provide a good description of the experimental data^4^. We also calculated electronic band structures to assess the evolution of the *s* and *d* bands under pressure.

## Supplementary information

Supplementary Information

## Data Availability

All the data generated in the study are available upon reasonable request to the corresponding authors.
