# Supplementary material for: Record high Tc element superconductivity achieved in titanium
Source: Nat Commun. 2022 Sep 15;13:5411. doi: 10.1038/s41467-022-33077-3 (PMC9478155; doi:10.1038/s41467-022-33077-3)
Supplement: Supplementary file 1 — Supplementary Information [file 41467_2022_33077_MOESM1_ESM.pdf]

Supplementary Information

Record High T_c Element Superconductivity Achieved in Titanium

Changling Zhang[§1,2], Xin He [§1,2,3], Chang Liu[§4], Zhiwen Li[1,2], Ke Lu[1,2], Sijia Zhang[1], Shaomin Feng[1], Xiancheng Wang*[1,2], Yi Peng[1,2], Youwen Long[1,2, 3], RichengYu[1,2], Luhong Wang[5], Vitali Prakapenka[6], Stella Chariton[6], Quan Li[4], Haozhe Liu[7], Changfeng Chen*[8], Changqing Jin*[1,2,3]

[1]*Beijing National Laboratory for Condensed Matter Physics, Institute of Physics, Chinese Academy of Sciences, Beijing 100190, China*

[2]*School of Physical Sciences, University of Chinese Academy of Sciences, Beijing 100190, China*

[3] *Songshan Lake Materials Laboratory, Dongguan 523808, China*

[4]*International Center for Computational Method and Software, College of Physics, Jilin University, Changchun 130012, China*

[5] *Shanghai Advanced Research in Physical Sciences, Shanghai 201203, China*

[6] *Center for Advanced Radiations Sources, University of Chicago, Chicago, Illinois 60637, USA*

[7] *Center for High Pressure Science & Technology Advanced Research, Beijing 100094, China*

[8] *Department of Physics and Astronomy, University of Nevada, Las Vegas, Nevada 89154, USA*

[§] These authors contributed equally to the paper

* Corresponding authors; wangxiancheng@iphy.ac.cn; changfeng.chen@unlv.edu; jin@iphy.ac.cn

Supplementary Methods

The electrical resistance and Hall effect measurements are performed using the four probe Van der Pauw method as shown in Supplementary Fig. 1a&b. The standard way is to use 6 probes to measure Hall signals. However concerning the 30μm sample space as well as tiny specimen(<10 μm) in Megabar pressure chamber four probes based on Van der Pauw method are sometimes applied. In our manuscript, the resistance and Hall coefficient are measured via the four probe Van der Pauw method, which is usually used for small samples with irregular and two dimensional shape [Supplementary Reference 1~3]. For all the samples in the experiments, the electrode and the sample configurations are the same as shown in Supplementary Fig. 1b. For resistance experiments, the electrodes of 1 and 4 can be used for applying current, and the electrodes of 2 and 3 for the longitudinal voltage measurement. While for Hall coefficient measurements, the electrodes of 1 and 3 are used for applying current, and the electrodes of 2 and 4 for the Hall voltage measurement as described by Van der Pauw method in Supplementary Reference 1.

The pressures are calibrated via the shift of the first-order Raman edge frequency from the diamond cutlet by the following equation[18]. There are two first-order Raman edges at low and high frequencies of 1344 cm^{-1} and 1735 cm^{-1}, which come from the table face (ambient pressure) and culet (high pressure) of the anvil, respectively.

$$P(\mathrm{GPa}) \cong K_0 \frac{\Delta\nu}{\nu_0}\left[1 + \frac{1}{2}(K_0' - 1)\frac{\Delta\nu}{\nu_0}\right] \quad (1)$$

Here, K_0 = 547 GPa and K_0’ = 3.75 are the bulk modulus and pressure derivative of bulk modulus of diamond, respectively. ν_0 and ν are the edge frequencies at ambient pressure and high pressure, respectively. $\Delta\nu$ is the difference between ν_0 and ν.

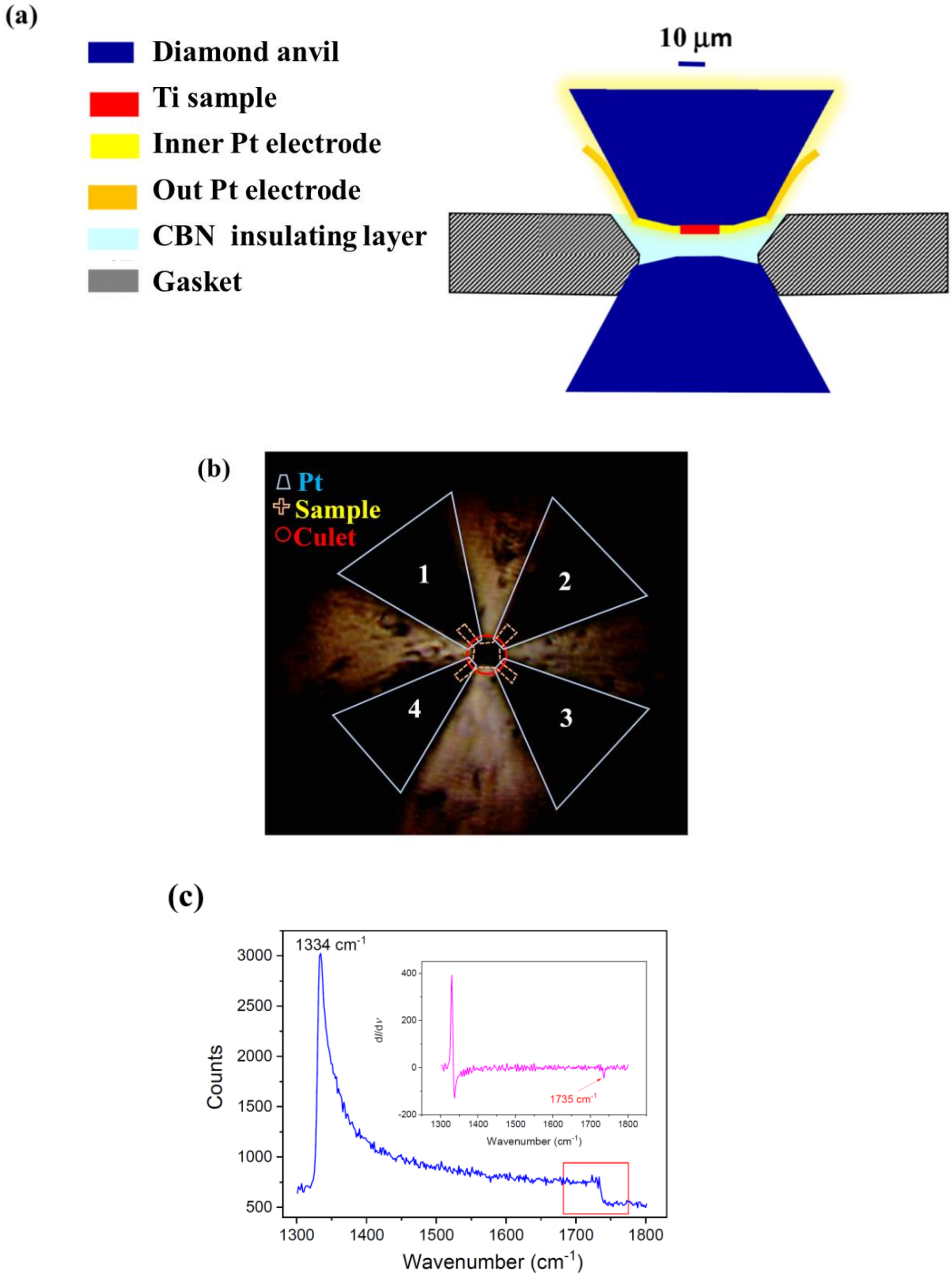

Supplementary Figure 1. (a) The schematic view of the high pressure assembly for diamond anvil cell. (b) The electrode configuration for four probes Van der Pauw method. (c) Typical Raman spectrum from the center of the diamond anvil culet at high pressure. The inset is the differential spectrum, clearly showing the Raman edge frequency.

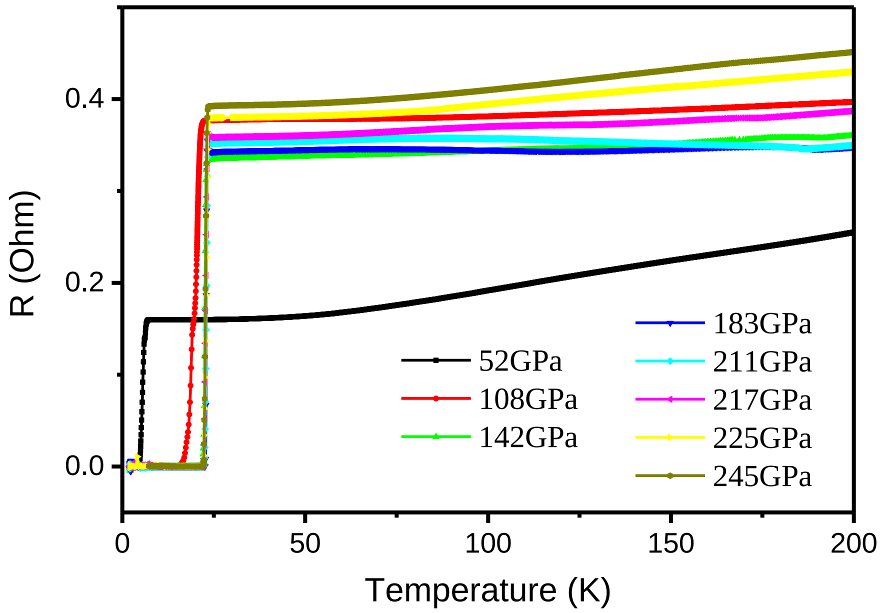

Supplementary Figure 2. The superconductivity measurements. Temperature dependence of the electrical resistance of elemental metallic Ti (Sample 5) measured at high pressures.

(a)

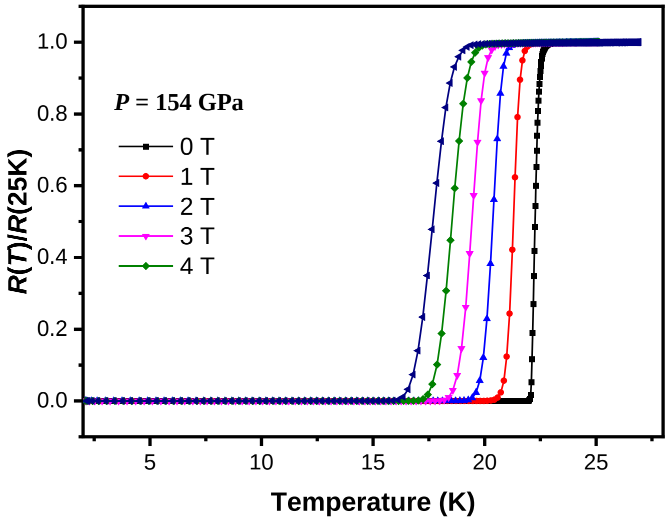

(b)

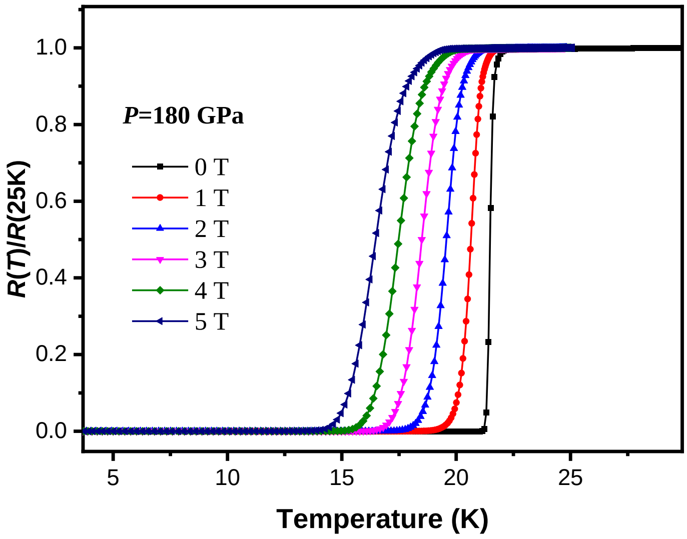

Supplementary Figure 3. The superconductivity under magnetic field. (a) The superconducting transitions measured at different magnetic fields at 154 GPa and (b) 180 GPa, respectively.

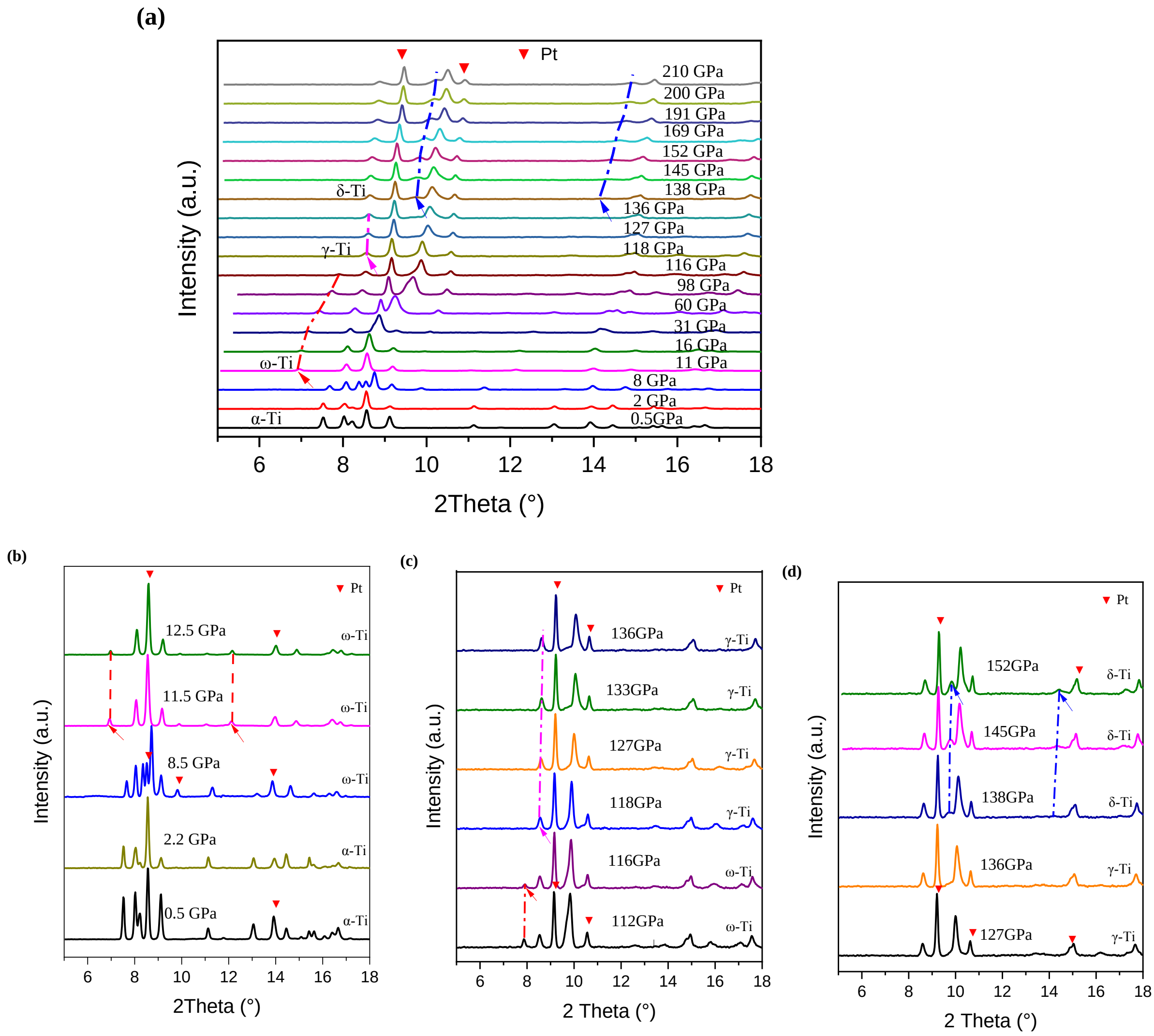

Supplementary Figure 4. The X-ray diffraction and phase transition. (a) The *in-situ* high pressure X-ray diffraction patterns with the highest experimental pressure of 210 GPa and the wavelength of 0.3344 Å. (b-d) The diffraction patterns with the pressure of 0.5-12.5 GPa, 112-136 GPa and 127-152 GPa, respectively. The Ti-α to Ti-ω phase transition at ~11.5 GPa can be evidenced with the appearance of two new peaks at 6.94 ° and 12.12 °. The disappearance of the peak at 7.90 ° above 116 GPa hints the Ti-ω to Ti-γ phase transition. The Ti-γ to Ti-δ phase transition occurs at 138 GPa, where two new peaks at ~9.72 ° and ~14.18 ° appear. The pressure induced phase transition sequence from Ti-α phase, to Ti-ω phase, to Ti-γ phase, and to Ti-δ phase has been observed, in consistent with the previously reported results [6].

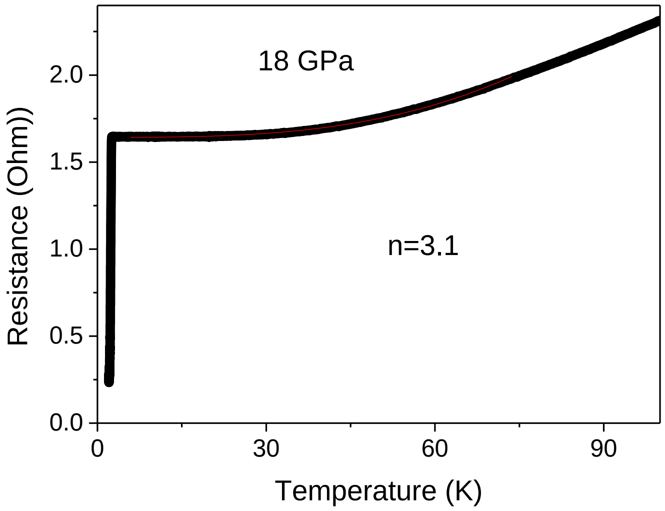

Supplementary Figure 5. The *s*-*d* interband scattering. The temperature dependence of resistance measured at 18 GPa. The resistance in the low temperature range of 6 to 70 K is fitted (red line) with the formula of $R= R_0+AT^n$, where R_0 is the residual resistance, A is the coefficient of the power law, and n=3.1 is the extracted exponent. The n value near 3 implies that the *s*-*d* interband scattering dominates the electron transport with contributions from electron correlation effects.

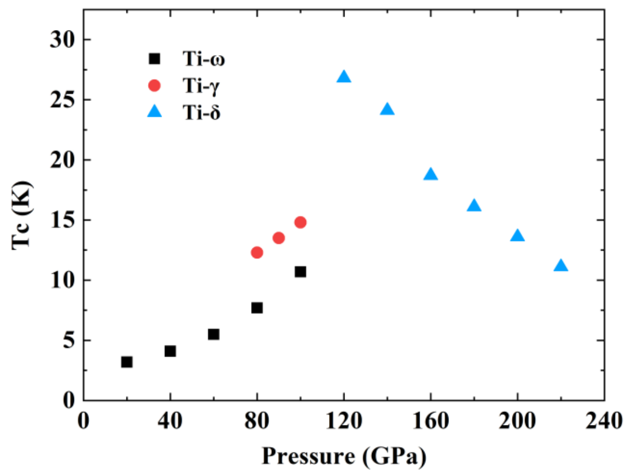

Supplementary Figure 6. The calculated T_c versus pressure. The superconducting critical transition temperature (T_c) of the indicated Ti phases in their stability range from 20 to 220 GPa hydrostatic pressures. The calculated crystal structure of Ti is sensitive to the pressure conditions, and under hydrostatic pressures at or above 160 GPa, the Ti-δ phase essentially relaxes to the Ti-β (bcc) phase without considering any stress anisotropy.

Supplementary References:

1. L. van der Pauw J., “A Method of Measuring Specific Resistivity and Hall Effect of Discs of Arbitrary Shape”, **Philips Research Reports 13**, 1(1958);

2. Errandonea, D. *et al.* “Investigation of conduction-band structure, electron scattering mechanisms, and phase transitions in indium selenide by means of transport measurements under pressure”, **Physical Review B 55**, 16217(1997);

3. Zhang, J. K. *et al*. “Electronic topological transition and semiconductor to metal conversion of Bi2Te3 under high pressure”, **Applied Physics Letters 103**, 52102(2013)].
